# Depressive Symptoms and Amygdala Volume in Elderly with Cerebral Small Vessel Disease: The RUN DMC Study

**DOI:** 10.4061/2011/647869

**Published:** 2011-10-06

**Authors:** I. W. M. van Uden, A. G. W. van Norden, K. F. de Laat, L. J. B. van Oudheusden, R. A. R. Gons, I. Tendolkar, M. P. Zwiers, F-E. de Leeuw

**Affiliations:** ^1^Department of Neurology, Donders Institute for Brain, Cognition and Behaviour, Radboud University Nijmegen Medical Centre, Reinier Postlaan 4, P.O. Box 9101, 6500 HB Nijmegen, The Netherlands; ^2^Department of Psychiatry, Donders Institute for Brain, Cognition and Behaviour, Radboud University Nijmegen Medical Centre, 6500 HB Nijmegen, The Netherlands; ^3^Donders Institute for Brain, Cognition and Behaviour, Centre for Cognitive Neuroimaging, Radboud University Nijmegen, Kappittelweg 29, 6525 EN, Nijmegen, The Netherlands

## Abstract

*Introduction*. Late onset depressive symptoms (LODSs) frequently occur in elderly with cerebral small vessel disease (SVD). SVD cannot fully explain LODS; a contributing factor could be amygdala volume. We investigated the relation between amygdala volume and LODS, independent of SVD in 503 participants with symptomatic cerebral SVD. *Methods*. Patients underwent FLAIR and T1 scanning. Depressive symptoms were assessed with structured questionnaires; amygdala and WML were manually segmented. The relation between amygdala volume and LODS/EODS was investigated and adjusted for age, sex, intracranial volume, and SVD. *Results*. Patients with LODS had a significantly lower left amygdala volume than those without (*P* = 0.02), independent of SVD. Each decrease of total amygdala volume (by mL) was related to an increased risk of LODS (OR = 1.77; 95% CI 1.02–3.08; *P* = 0.04). *Conclusion*. Lower left amygdala volume is associated with LODS, independent of SVD. This may suggest differential mechanisms, in which individuals with a small amygdala might be vulnerable to develop LODS.

## 1. Introduction

A first depressive episode at older age is defined as late onset depressive symptoms (LODSs). LODSs are usually defined by their occurrence after the age of 60 years, while early onset depressive symptoms (EODSs) appear before. The prevalence of LODS and EODS ranges from 10% to 32% [1–4] in the elderly population. Patients with EODS show a higher rate of family history of depression than patients with LODS; genetic factors appear to be important. While psychological and genetic factors presumably play an important role in EODS [5], there are probably other nongenetic factors that play a role in LODS [5, 6] such as structural changes in the brain [7, 8]. 

Population-based neuroimaging studies suggest a possible role for frequently occurring white matter lesions (WMLs) in the etiology of LODS [9–12]. Their dominant view is that WMLs disrupt white matter tracts connecting cortical and subcortical structures (e.g., frontostriatal circuits), which result in LODS [13, 14]. 

As WML are part of the cerebral small vessel disease (SVD) spectrum, also including lacunar infarcts, it could be that the association between WML and LODS is driven by lacunar infarcts as they reflect more severe structural damage than WML. In addition, SVD might not fully explain the presence of depressive symptoms in elderly as there are patients with LODS without SVD. 

A functional or structural change in the amygdala may explain the residual depressive symptoms as the amygdala is involved in mood regulation, and structural MRI studies showed a lower amygdala volume in patients with both LODS and EODS than in healthy controls [15–18]. However, these studies did not adjust for the possible confounding role of SVD. Conversely, none of the population-based studies on SVD and LODS took amygdala volume into account. We therefore wanted to investigate the relation between amygdala volume and the presence of depressive symptoms in patients with SVD, with adjustment for degree of SVD.

## 2. Patients and Methods

### 2.1. Study Population

The Radboud University Nijmegen Diffusion tensor and Magnetic resonance imaging Cohort (RUN DMC) study is a prospective cohort study that investigates the risk factors and cognitive, motor, and mood consequences of functional and structural brain changes among elderly with cerebral SVD. 

Cerebral SVD is characterised on neuroimaging by either WML or lacunar infarcts. Symptoms include acute symptoms, such as transient ischaemic attacks (TIAs) or lacunar syndromes, or subacute manifestations, such as cognitive, motor (gait) and/or mood disturbances [19]. As the onset of cerebral SVD is often insidious, clinically heterogeneous, and typically with mild symptoms, it has been suggested that the selection of subjects with cerebral SVD in clinical studies should be based on the more consistent brain imaging features [20]. 

Accordingly, in 2006, consecutive patients who visited the department of neurology, between October 2002 and November 2006, were selected for participation. Inclusion criteria were (a) age between 50 and 85 years; (b) cerebral SVD on neuroimaging (WML and/or lacunar infarcts). Subsequently the above-mentioned acute or subacute clinical symptoms of SVD were assessed by standardized structured assessments. Patients who were eligible because of a lacunar syndrome were included only >6 months after the event to avoid acute effects on the outcomes. 

Exclusion criteria were (a) dementia and (b) parkinson(ism) according to the international diagnostic criteria [21–23]; (c) life expectancy of less than six months; (d) intracranial space occupying lesion; (e) (psychiatric) disease interfering with cognitive testing or followup; (f) recent or current use of acetylcholine-esterase inhibitors, neuroleptic agents, L-dopa, or dopa-a(nta)gonists; (g) WML mimics (e.g., multiple sclerosis and irradiation induced gliosis); (h) prominent visual or hearing impairment; (i) language barrier; (j) MRI contraindications or known claustrophobia.

Patients were selected in a three-step approach. After reviewing medical records 1004 individuals were invited by letter, 727 were eligible after contact by phone, and 525 agreed to participate. In 22 subjects exclusion criteria were found during their visit to our research centre (14 with unexpected claustrophobia, 1 died before MRI scanning, 1 was diagnosed with multiple sclerosis, in 1 there was a language barrier, 1 subject fulfilled the criteria for Parkinson's disease, and 4 met the dementia criteria), yielding a response of 71.3% (503/705). From these 503 individuals one group had symptoms of TIA or lacunar syndrome (*n* = 219), and the remaining (*n* = 284) had cognitive disturbances, motor disturbances, depressive symptoms, or a combination thereof. All participants signed an informed consent form. The Medical Review Ethics Committee region Arnhem-Nijmegen approved the study.

### 2.2. MRI Acquisition

Imaging was performed on a 1,5 Tesla MRI scanner (Magnetom, Sonata; Siemens Medical Solutions, Erlangen, Germany). The protocol included 3D MPRAGE imaging (TR/TE/TI 2250/3.68/850 ms; flip angle15°; voxel size 1.0 × 1.0 × 1.0 mm) and fluid attenuated inversion recovery (FLAIR) sequences (TR/TE/TI 9000/84/2200 ms; voxel size 1.0 × 1.2 × 5.0 mm, with an interslice gap of 1.0 mm). All scans were performed on the same scanner.

### 2.3. Amygdala Volumetry

One investigator, blinded to the clinical and other imaging data (IvU), performed manual segmentation of the amygdala, using the interactive software program “ITK-SNAP” [24] (http://www.itksnap.org/). Briefly, this program allowed simultaneous viewing of volumes in coronal sagittal and transversal view, thereby permitting a neat handling of anatomical borders while segmenting the regions of interest. Left and right amygdalae were manually segmented in the coronal plane, from posterior to anterior. Next, segmentations were reviewed in the sagittal plane, because then boundaries were better visualized [25–27]. Segmentation was performed according to previously published protocols [27, 28], and the correct segmentation of anatomical boundaries was verified with the aid of neuroanatomical atlases [28, 29]. In short, the first slice of the amygdala, the posterior border, was identified superior to the hippocampus at the point where the white matter first starts to appear superior to the alveus and laterally to the hippocampal head. The anterior border of the amygdala was defined at the level where the amygdala no longer has an ovoid shape. The medial border is marked by the medial margin of the temporal lobe, which borders Cerebral Spinal Fluid (CSF). The lateral/inferior border is the surrounding white matter and the inferior horn of the lateral ventricle. The amygdala and hippocampus were carefully separated on the sagittal view, moving from the medial to the lateral side of the brain.

Segmentations were done in a standardized way by rating the left amygdala first for half of the patients and the right amygdala first for the other half. Volume was calculated for the left and right amygdala separately by summing all segmented areas, multiplied by slice thickness. Intrarater on a random sample yielded an intraclass correlation coefficient for both left and right amygdalae of 0.8.

### 2.4. WML Volumetry and Lacunar Infarcts

White matter signal hyperintensities on FLAIR scans, which were not, or only faintly hypointense on T1-weighted images, were considered WML, except for gliosis surrounding infarcts. WMLs were manually segmented on transversal FLAIR images, by 2 trained raters, (IvU, LvO), blinded for all clinical data and amygdala volumes. Total WML volume was calculated in the same fashion as for both amydalae. Inter-rater variability for total WML volume was determined on a random sample of ten percent yielded an intra-class correlation coefficient of 0.99. 

Lacunar infarcts were defined as areas with a diameter >2 mm and <15 mm with low signal intensity on FLAIR and T1, ruling out enlarged perivascular spaces and infraputaminal pseudolacunes [30]. Evaluation of infarcts was performed by one person with a good intra-rater variability with a weighted kappa of 0.80. In ten percent of the scans inter-rater variability was calculated with a weighted kappa of 0.88.

### 2.5. Brain Volumetry

Gray (GM), white matter (WM) tissue, and CSF probability maps were computed using SPM5 routines (Wellcome Department of Cognitive Neurology, University College London, UK). Total GM, WM, and CSF volumes were calculated by summing all voxel volumes that had a *P* > 0.5 for belonging to the tissue class. Intracranial volume (ICV) was taken as the sum of total GM, WM, and CSF.

### 2.6. Assessment of Depressive Symptoms

Depressive symptoms were assessed with the Center of Epidemiologic Studies Depression Scale (CES-D) [31]. Depressive symptoms were considered present in patients with a CES-D score ≥16 and/or current use of antidepressive medication, taken for depression, irrespective of their actual CES-D score, because depressive symptoms were considered to be the indication for the medication prescription.

In addition, all patients were asked about their history of depressive episodes. If depressive episodes had occurred, the patients were asked for the age of onset and whether the episodes had prompted them to seek medical advice. A history of depression was considered present if depressive episodes in the past had required attention of a general practitioner, psychologist, or psychiatrist [4]. 

According to the literature we used the age of 60 years as cut-off point to distinguish between LODS and EODS [4]. Patients at the age or older than 60 years with a CES-D ≥ 16 and/or current use of antidepressive medication, taken for depression, without a history of depressive episodes before or at the age of 60, were classified as having LODS. Individuals with a CESD < 16, without a history of depressive symptoms and without the current use of antidepressive medication, formed the reference group. All others fulfilled the criteria for EODS (first depressive episode <60 years).

### 2.7. Statistical Analysis

We compared the amygdala and WML volumes (overall, left, right) between the EODS and LODS group with the reference using ANCOVA. Adjustments for age, sex, ICV, WML (or amygdala volume with WML being dependent variable) and presence of lacunar infarcts were made. 

The risk of LODS and EODS per milliliter increases in amygdala volume, WML volume, and presence (yes/no) of lacunar infarcts was calculated (expressed as the odds ratio (OR) with a 95% confidence interval; 95% CI) by means of age, sex, ICV, adjusted logistic regression analysis with additional adjustment for the appropriate structural MRI measures (WML volume, amygdala volume, or lacunar infarcts). All data were analyzed using SPSS statistical software, version 16.0. *P* values < 0.05 were considered statistical significant.

## 3. Results

Of the 503 patients one was excluded because of an automatic segmentation problem that could not be solved manually. Two patients did not complete the CES-D questionnaire and of two patients the history of depression was not known. There were 101 individuals with LODS (20.3%) and 108 with EODS (21.7%); the reference group comprised 289 persons (58.0%).

Demographic and neuroimaging characteristics of 498 patients are shown in Table 1.

Mean age of the population was 65.6 years (SD 8.8), and 56.4% were male. Mean age of the LODS group was 71.4 years and of the EODS group 59.1 years. Mean age of onset of EODS was 45.1 years (SD 10.9).

 Total amygdala volume was 3.4 mL (SD 0.5), and mean volume of left amygdala was 1.8 mL (SD 0.3) and differed significantly (*P* < 0.001), from the right amygdala 1.6 mL (SD 0.3). Amygdala volume decreased significantly with age (*β* = − 0.339;   *P* < 0.001), and women had smaller amygdala (3.2 mL; SD 0.4) than men (3.5 mL; SD 0.5; *P* < 0.001). 

Table 2 shows that patients with LODS had a higher WML volume (21.8 mL; SD 20.2) than the reference group (14.7 mL; SD 18.0), although not significant (*P* = 0.06). Independent of SVD, patients with LODS had a significant lower left amygdala volume (1.6 mL; SD 0.3; *P* = 0.017) than the reference group (1.8 mL; 0.28); this difference was not found for the right amygdala (1.5 mL; SD 0.3, *P* = 0.432).

Patients with EODS did not differ from the reference group with respect to WML volume, amygdala volume, and the proportion of lacunar infarcts. We found no significant differences between left, right, and total amygdala volume, WML volume, and presence of lacunar infarcts between patients with LODS and EODS.

Table 3 shows the risk of LODS and EODS and per mL decrease in total, left, and right amygdala volume, WML volume (mL), and presence of lacunar infarcts. Each decrease of both total and left amygdala volume (mL) showed a significant increased risk of the presence of LODS (OR = 1.77; 95CI 1.02–3.08; *P* = 0.04, in total amygdala volume and OR 2.92; 95CI 1.22–7.01; *P* = 0.02 in left amygdala volume), independent of SVD. In addition, there was a nearly significant (*P* = 0.08) increased risk for LODS per increase of WML volume. This was not found for EODS and decrease of amygdala volume (OR = 0.95; 95CI 0.56–1.61; *P* = 0.86) per increase of WML volume, or presence of lacunar infarcts.

## 4. Discussion

In this study of 498 elderly patients with cerebral SVD, left amygdala volume was related to LODS, independent of WML volume and the presence of lacunar infarcts; this was not found for EODS and amygdala volume (left, right nor total). 

Before conclusions can be drawn, there are some methodological issues that need to be addressed. The proportion of patients with depressive symptoms in our study is relatively high (42%) for LODS and EODS together. In other studies the proportion of patients with depressive symptoms varies between 9.6%–32% [1–4, 32]. A possible explanation for our relatively high proportion of patients with depressive symptoms could be the fact that we purposely included patients on the basis of presence of SVD; consequently the median degree of WML volume in our study is higher than in population-based studies [33]. As these lesions are related to LODS it seems reasonable to expect a concomitant increased presence of depressive symptoms. Our finding of the (borderline significant) association between WML volume and LODS is in line with findings from these population-based studies [1, 4, 34]. Another explanation is that we classified patients as suffering from depressive symptoms once they had had a depressive episode in their medical history or when they used antidepressive drugs at baseline examination (while previous studies usually did not assess detailed information on the use of medication) [35]. The third explanation could be that we included patients with a history of lacunar stroke and transient ischaemic attacks. It is known that in this population the prevalence of depressive symptoms is higher compared to the general population [36]. 

To elucidate the etiological mechanisms of LODS its current widely used definition suffers from a conceptual problem, due to the fact that age during the first depressive episode determines the classification. It could very well be that recovery after EODS occurs while depressive symptoms develop again after sixty years of age. Despite the fact that these patients still fulfil the definition of EODS because of their history, they may have developed their LODS on the basis of another underlying pathology including SVD. This could have led to overrepresentation of WML among the EODS group. 

There is a conceptual problem with LODS and EODS. By definition the EODS sample included many subjects with major depression. All had consulted a doctor or were at one time treated with antidepressants. By contrast, the LODS sample was defined primarily by the CES-D score (and/or current antidepressant use). Some may have had major depression and some minor depression. These potential differences may affect results both ways.

Another limitation is the cross-sectional nature of our study, which prevents us from proving causality. The RUN DMC study has a longitudinal design, and followup is already planned to evaluate the effect of brain changes on depressive symptoms [37].

Strengths of our study include its design of a homogeneous population that covers the whole spectrum of cerebral SVD, its size and high response rate of over 70%, and the use of a single expert who segmented the amygdala, blinded to clinical information. Particularly the definition of the anatomic boundaries of the amygdala is a notorious problem in amygdala segmentation. Our use of one single, experienced rater minimized the effect of differential segmentation between several raters thereby limiting the effect misclassification. Our results are in line with those from meta-analyses on amygdala volume in nonclinical samples that found a mean volume of both left and right amygdala in 39 studies of 1.7 mL, with a range from 1.0 to 3.9 mL [28].

Although the amygdala is critical to the interpretation of emotion [16, 17] and implicated in mood disorders, volumetric studies of the amygdala in patients with mood disorders have provided inconsistent results [18]. Studies of chronic or recurrent depressive patients have found identical [38, 39], smaller [40–42] but also larger [43] amygdala volumes compared to controls. Postmortem studies showed a smaller amygdala in depressed patients compared to controls, probably due to fewer glial cells [15]. 

Our data showed a significant relationship with LODS and left amygdala volume; we did not find this relation with the volume of the right amygdala. This is concordant with previous neuroimaging and postmortem studies that have reported left lateralized atrophy in the prefrontal cortex and amygdala in mood disorders [15, 41, 44, 45]; however previous results are inconclusive as others also report on an association between a decrease in right amygdala volume and mood [40]. In addition some functional imaging studies have shown lateralization, with activation of the left amygdala, in relation to emotion and emotional information processing [46, 47].

A possible explanation for a smaller amygdala in patients with LODS could be the coexistent SVD, abnormalities in blood flow, metabolism, and neurotransmitter receptors [48, 49] which may, either directly or indirectly, lead to amygdala atrophy. Intact connectivity of the frontostriatal circuits is important in mood regulation [14, 50]. According to the “vascular depression” hypothesis [14] SVD, that tends to have a high prevalence in the frontostriatal regions, disrupts fiber tracts within these circuits, probably leading to depressive symptoms [11, 34].

There are two other studies that investigated the role of amygdala morphometry in LODS patients. One [51] showed that, despite insignificant amygdala volumetric findings, variations of amygdala shape can be detected and localized. They investigated so in 11 healthy elderly individuals and 14 depressed elderly individuals. A population-based cohort study found a relation between a history of depression, particularly early onset, and an increased risk of for Alzheimer's disease; however this risk was not mediated by smaller hippocampal or amygdala volumes at baseline [32]. In contrast to our results they did not find a relation between a history of depression or depressive symptoms at baseline and smaller amygdala volume. As discussed earlier, this could be because of the difference in degree of SVD between this population-based cohort and our study sample in which we included only subjects with some degree of SVD. As amygdala volume is related to the degree of SVD [52], our study sample will probably have smaller amygdalae. In addition there was a difference in WML segmentation, most studies used semiquantitative methods, and in contrast we manually segmented the WML volume. This in combination with the higher proportion of depressive symptoms in our study sample (42% versus 27%) can explain the relation we found between amygdala volume and LODS in contrast to the findings of this population-based cohort. Finally, the relation between LODS and amygdala volume in our cohort was mainly driven by the left amygdala, while this population-based cohort summed both amygdalae in their analysis. 

In conclusion, amygdala volume is associated with LODS, independent of SVD and not with EODS. Future research should consist of prospective studies in order to assess whether baseline presence of SVD increases the risk of amygdala atrophy at followup and whether this coincides with LODS. Innovative MRI techniques including diffusion tensor imaging (DTI) could offer promising tools in order to identify white matter tracts between the amygdala and other parts of the brain and the effects of SVD in those tracts with respect to the incidence of LODS.

##  Conflicts of Interests

The authors declare that there is no conflict of interests.

##  Authors' Contribution

The authors certify that all coauthors have contributed sufficiently to the writing of the paper. They have read and approved submission of the final version of the paper, and they have taken due care to ensure the integrity of the work.

## Figures and Tables

**Table 1:** Baseline Characteristics of patients with Late Onset Depressive Symptoms (LODSs), Early Onset Depressive Symptoms (EODSs), and patients without depressive symptoms.

Depressive symptoms	Late onset	Early onset	Reference	Overall
	(*n* = 101) (20.3%)	(*n* = 108) (21.7%)	(*n* = 289 ) (58.0%)	(*n* = 498) (100%)
Age yrs	71.4 (6.0)	59.1 (6.2)	65.9 (9.0)	65.6 (8.8)
Sex (male/female)	47/54	60/48	174/115	281/217
MSSE	27.4 (1.7)	28.4 (1.5)	28.3 (1.6)	28.1 (1.6)
Education (>primary school (%))	77.2%	95.4%	93.4%	90.6%
Mean CES-D	21.0 (8.0)	16.9 (11.0)	7.7 (5.0)	12.4 (9.3)
WM volume	438.4 (57.1)	484.7 (62.9)	465.3 (68.8)	464.1 (66.9)
GM volume	597.4 (66.7)	649.8 (60.5)	632.3 (65.8)	629.0 (67.1)
ICV	1660.8 (156.1)	1659.9 (146.5)	1687.9 (159.4)	1676.3 (156.3)
Median WML volume	16.8	4.5	8.0	8.0
Number of lacunar infarcts	40 (39.6%)	30 (27.8%)	101 (34.9%)	171 (34.3%)
Use of antidepressive medication	30 (29.7%)	30 (27.8%)	0 (0%)	60 (12.0%)

Number represent mean (SD) or number.

MMSE: Mini Mental State Examination; CES-D: Center for Epidemiological Studies Depression Scale; WM: white matter; GM: gray matter; ICV: intracranial volume; WML: white matter lesions.

**Table 2:** Adjusted mean amygdala volume, White Matter Lesions (WMLs) volume, and lacunar infarcts in patients with Late Onset Depressive Symptoms (LODSs), Early Onset Depressive Symptoms (EODSs), and patients without depressive symptoms.

	Patients with LODS (*n* = 101)	*P-*value^†^	Patients with EODS (*n* = 108)	*P-*value^†^	Reference group (*n* = 289)
Total amygdala volume (mL)*	3.2 (0.5)	0.05	3.5 (0.5)	0.82	3.4 (0.5)
Left amygdala (mL)*	1.6 (0.3)	0.02	1.8 (0.3)	0.71	1.8 (0.28)
Right amygdala (mL)*	1.5 (0.3)	0.43	1.7 (0.3)	0.90	1.6 (0.28)
WML volume (mL)**	21.8 (20.2)	0.06	9.5 (13.8)	0.94	14.7 (18.0)
Lacunar infarcts (%)***	32.9%	0.42	35.8%	0.47	32.0%

Number represent mean (SD) or number.

Data is shown of the comparison of LODS/EODS versus the reference group, *adjusted for age, sex, ICV, WML volume, and presence of lacunar infarcts, **adjusted for age, sex, ICV, presence of lacunar infarcts and amygdala volume, ***adjusted for age, sex, ICV, WML and amygdala volume, ^†^compared with the reference group.

**Table 3:** The risk of LODS and EODS per decrease in amygdala volume, white matter lesion (WML), volume and lacunar infarcts.

	Patients with LODS (*n* = 101)		Patients with EODS (*n* = 108)	
	OR	95% CI	OR	95% CI
Total amygdala volume*	1.77	1.02–3.08**^†^**	0.95	0.56–1.61
Left amygdala volume*	2.92	1.22–7.01**^†^**	0.98	0.41–2.30
Right amygdala volume*	1.53	0.59– 4.03	0.85	0.34–2.14
WML volume**	0.99	0.97–1.00	1.01	0.99–1.03
Lacunar infarcts***	1.32	0.78–2.23	0.80	0.46–1.39

Number represent odds ratio (OR), 95% confidence interval (CI), *adjusted for age, sex, WML, ICV volume, and presence of lacunar infarcts, **adjusted for age, sex, total amygdala volume, ICV, and presence of lacunar infarcts, ***adjusted for age, sex, ICV, WML, and amygdala volume, ^†^ *P* < 0.05.
